# Genome-Wide Identification and Analysis of the *AHL* Gene Family in Pepper (*Capsicum annuum* L.)

**DOI:** 10.3390/ijms26136527

**Published:** 2025-07-07

**Authors:** Xiao-Yan Sui, Yan-Long Li, Xi Wang, Yi Zhong, Qing-Zhi Cui, Yin Luo, Bing-Qian Tang, Feng Liu, Xue-Xiao Zou

**Affiliations:** 1 Key Laboratory for Vegetable Biology of Hunan Province, Engineering Research Center for Horticultural Crop Germplasm Creation and New Variety Breeding, Ministry of Education, College of Horticulture, Hunan Agricultural University, Changsha 410128, China; xiaoyan_sui@163.com (X.-Y.S.); yanlongli@hunau.edu.cn (Y.-L.L.); wxi759133669@163.com (X.W.); zuoyingtu@163.com (Y.Z.); cuiqz@stu.hunau.edu.cn (Q.-Z.C.); bqtang@126.com (B.-Q.T.); 2 Yuelushan Laboratory, Changsha 410128, China; luoyin4338@163.com

**Keywords:** *Capsicum annuum*, *AT-hook motif nuclear-localized* (*AHL*) gene family, gene duplication, floral development, stress response

## Abstract

*AT-hook motif nuclear-localized* (*AHL*) genes play critical roles in chromatin remodeling and gene transcription regulation, profoundly influencing plant growth, development, and stress responses. While *AHL* genes have been extensively characterized in multiple plant species, their biological functions in pepper (*Capsicum annuum* L.) remain largely uncharacterized. In this study, we identified 45 *CaAHL* genes in the pepper genome through bioinformatics approaches. Comprehensive analyses were conducted to examine their chromosomal distribution, phylogenetic relationships, and the structural and functional features of their encoded proteins. Phylogenetic clustering classified the CaAHL proteins into six distinct subgroups. Transcriptome profiling revealed widespread expression of *CaAHL* genes across diverse tissues—including roots, stems, leaves, flowers, seeds, pericarp, placenta, and fruits—at various developmental stages. Quantitative real-time PCR further demonstrated that *CaAHL1*, *CaAHL33*, and *CaAHL23* exhibited consistently high expression throughout flower bud development, whereas *CaAHL36* showed preferential upregulation at early bud development stages. Expression profiling under hormone treatments and abiotic stresses indicated that *CaAHL36* and *CaAHL23* are auxin-inducible but are repressed by ABA, cold, heat, salt, and drought stress. Subcellular localization assays in *Nicotiana benthamiana* leaf epidermal cells showed that both CaAHL36 and CaAHL23 were predominantly localized in the nucleus, with faint expression also detected in the cytoplasm. Collectively, this study provides foundational insights into the *CaAHL* gene family, laying the groundwork for future functional investigations of these genes in pepper.

## 1. Introduction

*Capsicum annuum* L., commonly known as chili pepper, is a globally significant crop renowned for its distinctive pungency and rich nutritional profile. Its economic importance is underscored by its extensive cultivation and the diverse products derived from it, including fresh produce, spices, and pharmaceuticals. Advancements in genomic research, notably the sequencing of the pepper genome, have paved the way for in-depth studies into gene families that influence key agronomic traits, thereby enhancing breeding programs aimed at improving yield, disease resistance, and stress tolerance [1,2,3].

The *AT-hook motif nuclear-localized* (*AHL*) gene family is defined by the presence of an AT-hook motif, a small DNA-binding domain that specifically interacts with AT-rich DNA regions, as well as a conserved plant and prokaryote conserved (PPC) domain. Over the past decade, the role of *AHL*s in regulating various plant growth and developmental processes has been increasingly recognized. These processes include the elongation of hypocotyls [4,5,6], the formation of pollen walls and flower development [7,8], root growth [9,10], petiole elongation [11], and the modulation of phytohormone signaling [11]. Furthermore, *AHL* genes have been implicated in plant responses to pathogen infections, as well as to environmental stresses such as salt and drought [12,13,14,15]. Recent studies have also broadened our understanding of the *AHL* gene family in important crops like cotton, soybean, maize, and rice [16,17,18,19]. These findings emphasize the diverse roles of *AHL*s in plant development, offering new strategies for crop improvement.

In recent years, significant progress has been made in understanding the roles of specific *AHL* genes in plant flower development and male sterility. In *Arabidopsis thaliana*, *AHL22* was identified as a critical regulator of flowering time by modulating *FLOWERING LOCUS T* (*FT*) chromatin state [20]. Similarly, *AHL16* suppresses transposon activation and ensured timely flowering [21]. *TRANSPOSABLE ELEMENT SILENCING* VIA *AT-HOOK* (*TEK*), also known as *AtAHL16*, was found to be essential for male fertility. *TEK* regulates arabinogalactan-proteins (*AGPs*) in anthers critical for pollen wall formation. Mutations cause microspore development defects and sterility [22]. *AHL15* promotes somatic embryogenesis, highlighting its biotechnological potential [23]. In rice, *PERSISTENT TAPETAL CELL2* (*PTC2*) regulates tapetal programmed cell death and pollen wall patterning, with its loss leading to male sterility [8]. However, despite these advancements, comprehensive investigations into the *AHL* gene family in chili pepper remain scarce.

Despite these advances, *AHL* genes in chili pepper remain largely unexplored. Given the importance of pepper and the potential roles of *AHLs* in development and stress adaptation, a genome-wide analysis of the pepper *AHL* family is warranted. Here, we identify and characterize all *CaAHL* (*Capsicum annuum AHL*) genes in the pepper genome and analyzed their expression patterns across different tissues and developmental stages, as well as in response to exogenous hormone treatments and abiotic stress conditions. In particular, we examine the expression of several *CaAHL* genes during floral development, providing insights into their potential roles in reproductive processes. The comprehensive data generated in this study lay the foundation for future functional analyses and may inform molecular breeding strategies aimed at improving pepper cultivars through the manipulation of *AHL* genes.

## 2. Results

### 2.1. Identification and Basic Information on the CaAHL Gene Family

The Hidden Markov Model (HMM) profile of the PPC domain (PF03479) was used as a query sequence to identify *AHL* proteins in the Zhangshugang genome database [24] using the “Simple HMM Search” function in TBtools-II (v2.149). In total, 45 *AHL* genes containing the conserved PPC domain were identified. Only those showing consistent hits in both the “Sequence scores” and “Domain scores” outputs were retained to ensure domain completeness and sequence reliability. To facilitate subsequent studies, these pepper *AHL* genes were designated as *CaAHL1* to *CaAHL45* based on their positions across the 12 chromosomes in the Zhangshugang reference genome. Comprehensive details, including gene names, chromosomal locations, protein lengths, molecular weights, theoretical isoelectric points (pIs), instability indices, grand average of hydropathicity, and predicted subcellular localizations, are provided in Table 1.

The *CaAHLs* exhibited significant variation in protein length and physicochemical properties. The protein lengths ranged from 111 amino acids (CaAHL24) to 578 amino acids (CaAHL39). Correspondingly, the molecular weights (MWs) varied from 11.71 kDa (CaAHL40) to 60.51 kDa (CaAHL39). The theoretical isoelectric points (pIs) spanned from 4.44 (CaAHL34) to 11.25 (CaAHL40), while the instability indices ranged from 30.43 (CaAHL24) to 63.62 (CaAHL31). The grand average of hydropathicity (GRAVY) values ranged from −0.76 (CaAHL4) to 0.18 (CaAHL27). Subcellular localization predictions for the 45 CaAHL proteins revealed diverse distribution patterns: 20 localized to the nucleus, 17 to the chloroplast, 5 to the cytoplasm, and 1 each to the plasma membrane, vacuole membrane, and endoplasmic reticulum (ER) (Table 1).

### 2.2. Chromosomal Distribution of the CaAHL Gene Family

**Figure 1:**
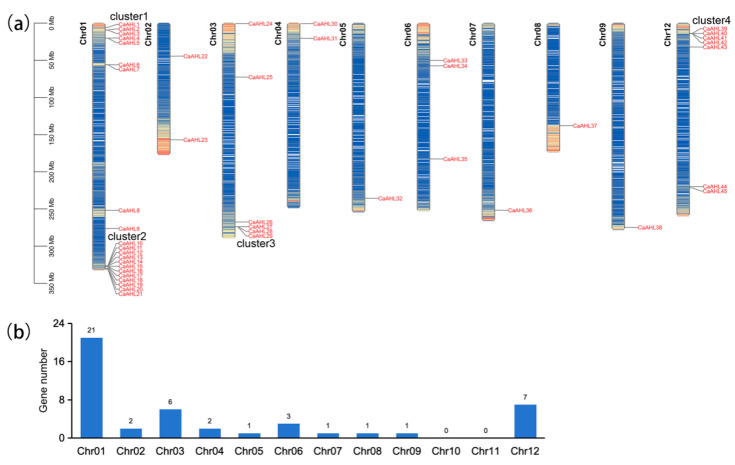
Chromosome mapping and gene number of *CaAHLs* in Zhangshugang genome. (**a**) Chromosome mapping of *CaAHLs*. Chromosome numbers are represented on the top, and the scale is shown on the left. (**b**) Statistical analysis of *CaAHLs* on the 12 chromosomes.

The 45 *CaAHLs* were unevenly distributed across 9 of the 12 chromosomes in the Zhangshugang genome (Figure 1a). Most *CaAHLs* were concentrated near the distal ends of the chromosomes. Only a few were located in central regions. Chromosome 1 had the highest number of *CaAHLs*, with a total of 21, followed by chromosomes 12 and 3, which contained 7 and 6 genes, respectively. In contrast, no *CaAHLs* were identified on chromosomes 10 and 11 (Figure 1b).

Interestingly, *CaAHLs* were enriched in four specific regions of the genome: the proximal and distal ends of chromosome 1, the distal end of chromosome 3, and the proximal end of chromosome 12. These regions were designated as cluster 1, cluster 2, cluster 3, and cluster 4, respectively. Cluster 1 comprises five genes (*CaAHL1* to *CaAHL5*), cluster 2 contains twelve genes (*CaAHL10* to *CaAHL21*), cluster 3 includes three genes (*CaAHL27* to *CaAHL29*), and cluster 4 consists of four genes (*CaAHL39* to *CaAHL42*) (Figure 1a). The uneven distribution and clustering of *CaAHLs* suggest gene accumulation may have occurred through tandem duplication events in specific genomic regions.

### 2.3. Phylogenetic Analysis of the CaAHL Gene Family

**Figure 2:**
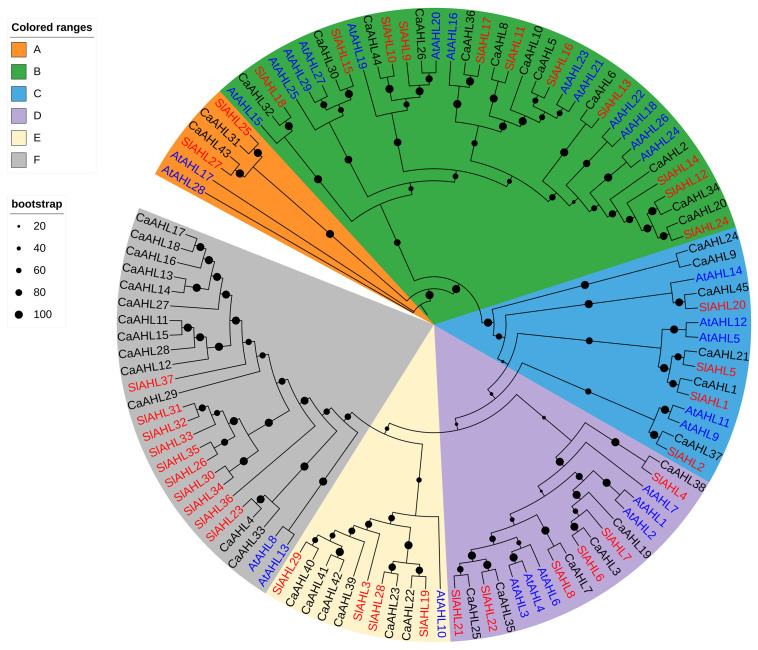
Phylogenetic relationships among *AHLs* in *Capsicum annuum*, *Solanum lycopersicum*, and *Arabidopsis thaliana*. Clades are shaded in distinct colors to facilitate visual differentiation. Black dots indicate bootstrap support values, with only those greater than 20 displayed.

To further delineate the evolutionary pathway of *AHL* genes across diverse species, a phylogenetic tree was constructed based on 45 pepper AHL amino acid sequences, along with those from tomato and *Arabidopsis thaliana* (Figure 2). The phylogenetic analysis revealed that the *CaAHL* gene family is divided into six main clades: Clade A, containing 2 genes; Clade B, containing 12 genes; Clade C, containing 6 genes; and Clade D, containing 6 genes; Clade E, containing 6 genes; Clade F, containing 13 genes. Interestingly, the number of *AHL* genes in pepper, tomato, and *Arabidopsis thaliana* shows only minor differences in Clade A (2, 2, and 2, respectively), Clade B (12, 11, and 13, respectively), Clade C (6, 4, and 5, respectively), and Clade D (6, 6, and 6, respectively) (Figure 2 and Table S1). However, more pronounced differences were observed in Clade E and Clade F. The number of *AHL* genes in Clade E is six, four, and one in pepper, tomato, and *Arabidopsis thaliana*, respectively. In Clade F, the corresponding values are 13, 10, and 2—indicating a notably higher number of *AHL* genes in pepper and tomato compared to *Arabidopsis thaliana* (Figure 2 and Table S1). Combined with chromosome localization analysis (Figure 1), we found that *CaAHL11*–*CaAHL18* in gene cluster 2 and *CaAHL27*–*CaAHL29* in gene cluster 3 are located in Clade E. Similarly, *CaAHL39*–*CaAHL42* in gene cluster 4 are positioned in Clade F. These results suggest that during the evolutionary process in the Solanaceae family, the *AHL* genes in Clade E and Clade F may have undergone duplication events.

### 2.4. Analysis of Conserved CaAHL Motifs

**Figure 3:**
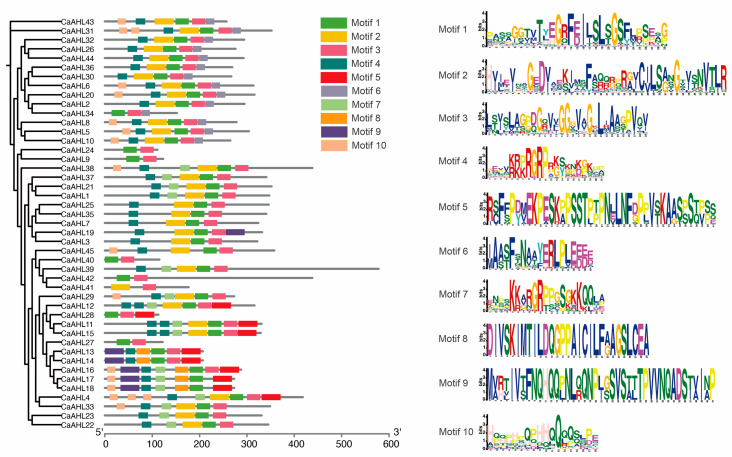
Analysis of conserved CaAHL motifs. The evolutionary tree on the left is constructed based on the protein sequences of CaAHLs.

We analyzed the motifs of the *CaAHL* genes and predicted their functions (Figure 3 and Table S1). The results indicated that most CaAHLs contained Motifs 1, 2, 3, and 4. Nearly all CaAHLs included Motif 1 and Motif 3, except for CaAHL41, which lacked Motif 1, and CaAHL17 and CaAHL18, which lacked Motif 3. Additionally, 82.22% and 73.33% of CaAHLs contained Motif 4 and Motif 2, respectively (Figure 3 and Table S2). Some CaAHLs contained multiple identical motifs. For example, CaAHL11, CaAHL12, and CaAHL15 each contained two Motif 4 elements. CaAHL31 and CaAHL4 contained two and three Motif 10 elements, respectively. We also found that CaAHL11 and CaAHL15; CaAHL13 and CaAHL14; as well as CaAHL23 and CaAHL24, exhibited highly similar motif structures, which is consistent with their clustering in the phylogenetic analysis (Figure 3 and Table S2). The four genes in cluster 4 exhibit significant differences in both motif number and protein length. This suggests that they are not tandem duplicates (Figure 3 and Table 1). However, the five genes in cluster 2 (CaAHL13, CaAHL14, CaAHL16, CaAHL17, and CaAHL18) exhibit high similarity in both gene structure and protein length. Notably, CaAHL16 contains Motif 3, Motif 7, and Motif 10, which are absent in CaAHL13 and CaAHL14, and possesses Motif 3, which is missing in CaAHL17 and CaAHL18. Overall, CaAHLs in the same subgroup in the phylogenetic tree tended to have similar structures and conserved motif distributions (Figure 3 and Table S2), indicating that CaAHLs contain highly conserved amino acid residues and that CaAHLs in the same cluster may have similar roles.

### 2.5. Cis-Regulatory Element Analysis of the CaAHL Promoters

**Figure 4:**
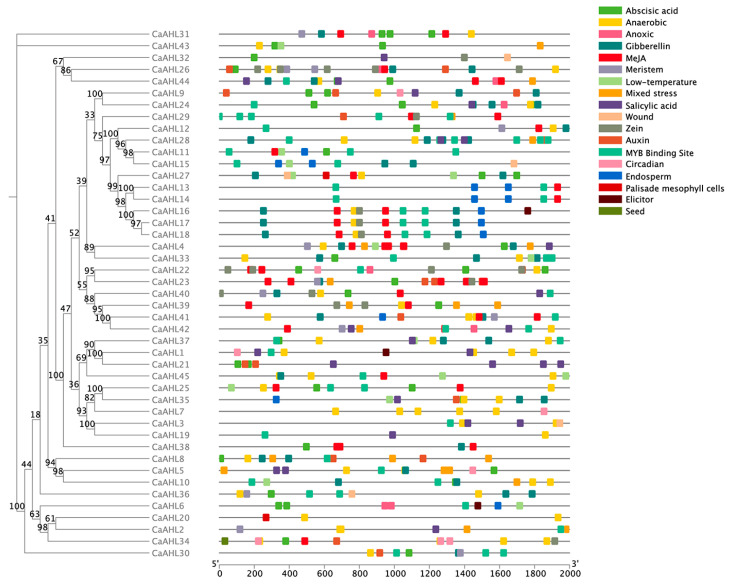
The distribution of cis-regulatory elements predicted in the *CaAHL* promoters. Different colored boxes represent different cis-regulatory elements. The evolutionary tree on the left is constructed based on the protein sequences of *CaAHLs*.

**Figure 5:**
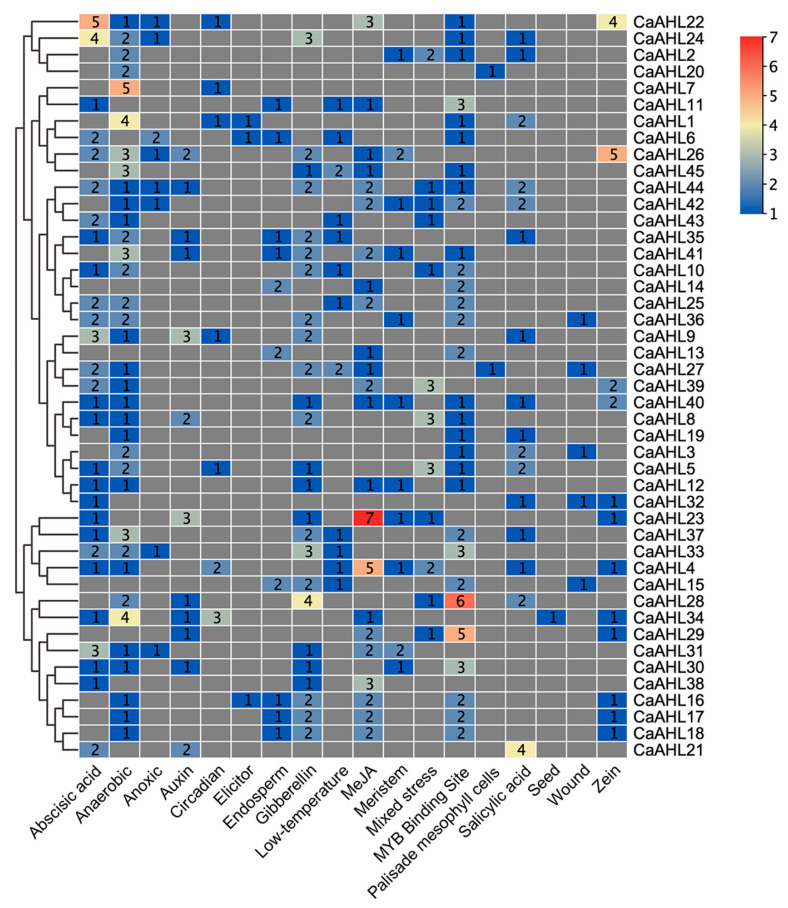
Number of cis-regulatory elements in the *CaAHL* promoter regions. The evolutionary tree on the left is based on the analysis of the number and types of cis-regulatory elements.

Since *CaAHLs* play important roles in the response to plant growth, development, and stress responses, we utilized PlantCARE to analyze the cis-regulatory elements in the pre-2000 bp region of the *CaAHLs* promoter to explore their possible functions. We screened cis-regulatory elements associated with growth, development, stress, and hormone responses. These were identified in the promoter regions of *CaAHLs* and categorized into 18 distinct classes using TBtools (Figure 4 and Figure 5). The analysis revealed that the promoters of *CaAHL22* and *CaAHL24* are enriched with abscisic acid-responsive cis-regulatory elements, containing five and four elements, respectively. Promoters of *CaAHL7*, *CaAHL1*, and *CaAHL34* exhibit a higher abundance of anaerobic-responsive elements, with five, four, and four elements, respectively. The promoter of *CaAHL28* shows a significant presence of gibberellin-responsive elements. Similarly, the promoters of *CaAHL23* and *CaAHL4* are enriched with jasmonic acid-responsive elements, containing seven and five elements, respectively. *CaAHL28* and *CaAHL29* have higher numbers of MYB binding site-related elements, with six and five elements, respectively. The promoter of *CaAHL21* contains four salicylic acid-responsive elements (Figure 4 and Figure 5). These distinct distributions of cis-regulatory elements suggest potential functional differentiation across the *CaAHLs*.

### 2.6. Tissue-Specific Expression Profiles of CaAHLs in Pepper

**Figure 6:**
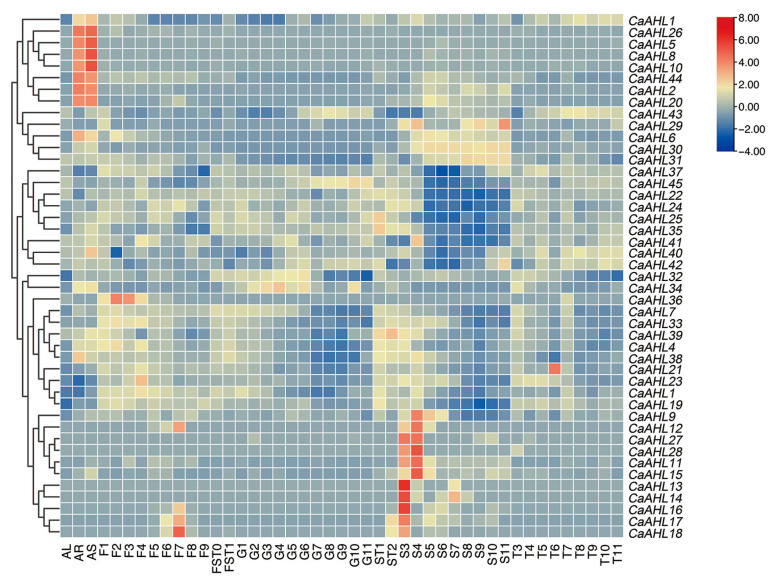
Expression profile analysis of *CaAHLs* in various tissues and organs of pepper. Expression levels were determined in the following tissues and stages: leaf tissues were sampled 60 days after emergence and marked correspondingly as AL; stems and roots were marked AS and AR, respectively. Floral buds were sampled at 0.25, 0.35, 0.5, 0.7, 0.8, 1.0, 1.2, 1.45, and 1.7 cm and marked correspondingly as F1, F2, F3, F4, F5, F6, F7, F8, and F9; fruits were collected 3, 7, 10, 15, 20, 25, 30, 35, 40, 45, 50, 55, and 60 days after flowering (DAF) and marked correspondingly as FST0, FST1, G1, G2, G3, G4, G5, G6, G7, G8, G9, G10, G11; seed samples were collected 10, 15, 20, 25, 30, 35, 40, 45, 50, 55 and 60 DAF and marked correspondingly as ST1, ST2, S3, S4, S5, S6, S7, S8, S9, S10, S11; placenta samples were collected 20, 25, 30, 35, 40, 45, 50, 55 and 60 DAF and marked correspondingly as T3, T4, T5, T6, T7, T8, T9, T10, and T11. The FPKM values were log2-transformed, and a heatmap was generated using TBtools-II (v2.149) software. The evolutionary tree on the left is constructed based on gene expression levels. Expression values on the right are shown as a color gradient from low expression (blue) to high expression (red).

To explore whether *CaAHLs* play a role in tissue development in pepper, the expression profiles of *CaAHLs* in the roots, stems, leaves, pericarp, and placenta were analyzed using RNA-seq data from the pepper line 6421 (Figure 6 and Table S3). The results revealed that several genes, including *CaAHL1*, *CaAHL26*, *CaAHL5*, *CaAHL8*, *CaAHL10*, *CaAHL44*, *CaAHL2*, and *CaAHL20*, exhibited root- and stem-specific expression (Figure 6). Certain genes, such as *CaAHL9*, *CaAHL27*, *CaAHL28*, and *CaAHL11* to *CaAHL18*, were more highly expressed in seeds at 20 and 25 days after flowering (DAF). Some genes were specifically expressed in certain tissues or developmental stages. For instance, *CaAHL29* displayed high expression in seeds at 60 DAF, suggesting a potential role in seed maturation. *CaAHL36*, *CaAHL23*, *CaAHL12*, and *CaAHL16* to *CaAHL18* were highly expressed during various stages of bud development, indicating their involvement in floral development. *CaAHL21* showed specific expression in the placenta at 35 DAF, implying a role in placenta development (Figure 6). These findings suggest that most *CaAHL* genes are specifically expressed in roots, stems, and seeds at 20 and 25 DAF, highlighting their significant roles in root, stem, and seed development. The tissue-specific expression patterns of certain genes indicate functional differentiation across the *CaAHL* family members.

### 2.7. Expression Profiles of CaAHL Genes in Response to Exogenous Hormones and Abiotic Stresses

The transcriptome data used for analyzing hormone and abiotic stress responses were derived from a previously published dataset [25], where RNA-seq results were experimentally validated by qRT-PCR, confirming their reliability for downstream expression analysis.

**Figure 7:**
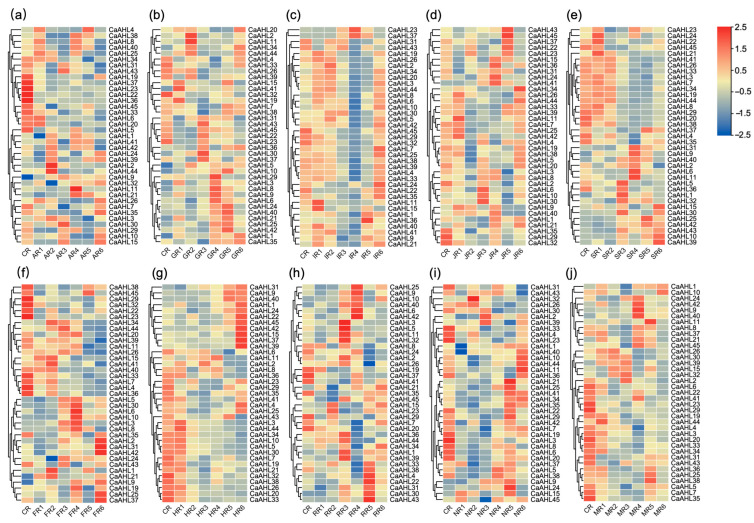
Expression profiles of *CaAHL* genes under exogenous hormones and abiotic stresses. Red blocks denote genes with high expression levels, while blue blocks represent genes with low expression levels based on transcriptomic data. All samples were collected from roots, with three biological replicates per treatment. Each treatment group includes a corresponding untreated control sample (CR: control root). (**a**) AR: ABA-treated root; (**b**) GR: GA3-treated root; (**c**) IR: IAA-treated root; (**d**) JR: JA-treated root; (**e**) SR: SA-treated root; (**f**) FR: cold-treated root; (**g**) HR: heat-treated root; (**h**) RR: H_2_O_2_-treated root; (**i**) NR: NaCl-treated root; (**j**) MR: mannitol-treated root. Numbers 1 to 6 represent time points after treatment, 0.5 h, 1 h, 3 h, 6 h, 12 h, and 24 h, respectively.

In this study, heatmap visualization was utilized to analyze the expression profiles of all *CaAHL* genes in pepper roots under the treatments of plant hormones and abiotic stresses (Tables S4 and S5). Root tissues were subjected to five plant hormones—abscisic acid (ABA), gibberellin (GA), indole-3-acetic acid (IAA), jasmonic acid (JA), and salicylic acid (SA)—and five abiotic stresses, namely low-temperature stress, high-temperature stress, simulated drought stress (mannitol), salt stress, and oxidative stress (hydrogen peroxide). Notably, eight *CaAHL* genes (*CaAHL12*, *CaAHL13*, *CaAHL14*, CaAHL16, *CaAHL17*, *CaAHL18*, *CaAHL27*, and *CaAHL28*) were undetectable in root tissues before and after all hormone and abiotic stress treatments, suggesting that these genes are not expressed in roots. Following different hormone and abiotic stress treatments, the detectable *CaAHL* genes in roots exhibited varying degrees of upregulation or downregulation, indicating divergent functional roles among these genes (Figure 7).

Among the six genes that were highly expressed during floral bud development (Figure 6), only *CaAHL23* and *CaAHL36* were detectable in roots. Both genes were significantly downregulated by abscisic acid (ABA) treatment (Figure 7a). Under gibberellin treatment, *CaAHL36* expression followed a dynamic pattern: it first decreased, then increased, then decreased again, and finally increased (Figure 7b). In contrast, *CaAHL23* expression initially decreased, then increased, and subsequently decreased again (Figure 7b). Following IAA treatment, *CaAHL23* and *CaAHL36* transcript levels peaked at 6 and 12 h, respectively (Figure 7c). Interestingly, under JA treatment, the peak expression times of these genes were reversed: *CaAHL23* peaked at 12 h and *CaAHL36* at 6 h (Figure 7d). Under SA treatment, *CaAHL23* expression showed fluctuations (increased, then decreased, then increased, and finally decreased), whereas *CaAHL36* showed an initial decrease, followed by an increase and a subsequent decrease (Figure 7e). Under abiotic stresses (cold, heat, salt, and mannitol-induced drought), the expression of both genes generally decreased, consistent with the downregulation observed in response to ABA (Figure 7a,f,g,i,j). These results suggest that *CaAHL23* and *CaAHL36* may play critical roles in ABA-mediated stress responses. However, under oxidative stress, both genes displayed a transient expression pattern with an initial increase, followed by a decrease and a subsequent increase (Figure 7h).

### 2.8. Relative Expression Levels of Eight CaAHL Genes in Bud Development

**Figure 8:**
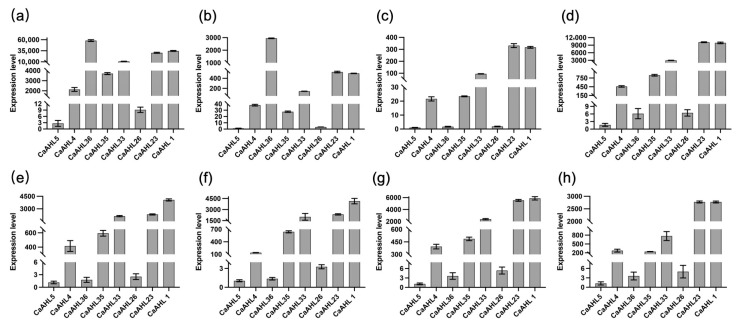
Relative expression levels of eight *CaAHL* genes in bud development. Floral buds were sampled at 0.25, 0.35, 0.5, 0.7, 0.8, 1.0, 1.2, and 1.45 cm and marked correspondingly as F1 (**a**), F2 (**b**), F3 (**c**), F4 (**d**), F5 (**e**), F6 (**f**), F7 (**g**), and F8 (**h**).

Based on the transcriptomic data from different stages of flower bud development (Figure 6), we observed that eight CaAHL genes—*CaAHL1*, *CaAHL4*, *CaAHL5*, *CaAHL23*, *CaAHL26*, *CaAHL33*, *CaAHL35*, and *CaAHL36*—exhibited detectable expression at one or more stages. To gain a preliminary understanding of their potential involvement in floral development, we conducted qRT-PCR to examine their expression patterns during these stages (Figure 8). Expression levels were normalized using *CaAHL5*, which showed the lowest transcript abundance across all stages, as the reference. The qRT-PCR results revealed that *CaAHL1*, *CaAHL4*, *CaAHL23*, *CaAHL33*, and *CaAHL35* consistently exhibited relatively high expression levels compared with *CaAHL5* during all stages of floral development (Figure 8), suggesting their sustained roles throughout flower formation. Using *CaAHL5* as the calibrator gene, we found that CaAHL*26* consistently exhibited a slightly higher relative expression than CaAHL*5* across most developmental stages, except at F2 and F3, where its relative expression was only marginally higher. Likewise, CaAHL*36* showed relatively high expression compared to CaAHL*5* at F1 and F2 (Figure 8). This suggests that *CaAHL36* may be specifically involved in the early phase of floral bud initiation or organ differentiation. These findings indicate that while some *CaAHL* genes are broadly involved in flower development, others may function in a stage-specific manner, reflecting possible sub-functionalization within the gene family.

### 2.9. Subcellular Localization of CaAHL23 and CaAHL36

**Figure 9:**
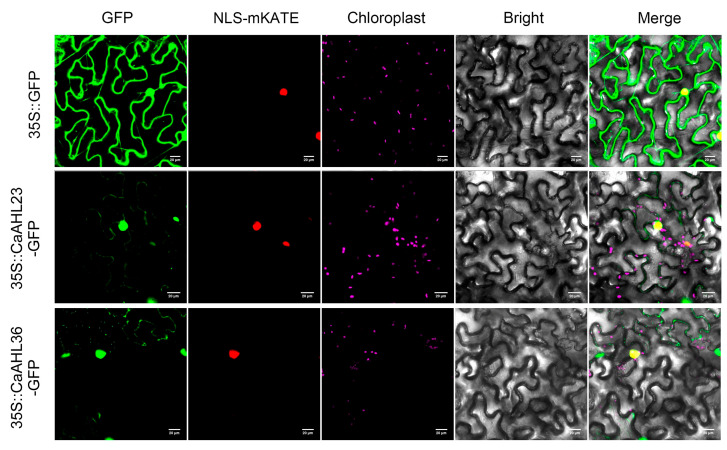
Subcellular localization of CaAHL23 and CaAHL36. The vectors 35S::CaAHL23-GFP, 35S::CaAHL36-GFP, and 35S::GFP were separately co-transformed into *Agrobacterium* with the nuclear localization marker NLS-mKATE. GFP: green fluorescent protein fluorescence signal. Chloroplast: chlorophyll autofluorescence signal. Bright: field of bright light; Merged: overlay of the GFP and DAPI signals, nuclear localization signal, chlorophyll autofluorescence signal, and bright light field. Scale bar = 20 μm.

To further investigate the cellular characteristics of CaAHL23 and CaAHL36, we conducted subcellular localization assays by fusing their coding sequences to GFP and transiently expressing them in *Nicotiana benthamiana* epidermal cells. The recombinant constructs (CaAHL23-GFP and CaAHL36-GFP) were driven by the CaMV 35S promoter, and infiltration was performed via *Agrobacterium tumefaciens*-mediated transformation. At 48 h post-infiltration, green fluorescence signals were detected using confocal laser scanning microscopy. The results showed that strong GFP fluorescence was predominantly localized in the nucleus for both CaAHL23 and CaAHL36 (Figure 9), consistent with their putative roles as transcription factors. Additionally, faint GFP signals were also observed in the cytoplasm, suggesting that a small proportion of the proteins may shuttle between the nucleus and cytoplasm or exhibit partial cytoplasmic distribution under these transient expression conditions. These findings provide preliminary evidence for the nuclear localization of CaAHL23 and CaAHL36 proteins, which is in line with their sequence-predicted function. However, further in vivo or stable expression studies would be necessary to confirm the dynamics and functional implications of their subcellular distribution.

## 3. Discussion

Our genome-wide analysis identified 45 *CaAHL* genes in pepper, substantially more than the 29 *AHLs* in *Arabidopsis* or the 35–40 typically found in other diploid plants [17,18,19,26]. This expansion likely reflects a history of gene duplication. The uneven chromosomal distribution and presence of four *CaAHL* clusters suggest that tandem duplications contributed to family expansion. Indeed, phylogenetic mapping reveals that the tandem arrays on chromosomes 1, 3, and 12 correspond to lineage-specific expansions in *Solanaceae*. Comparative phylogeny (Figure 2) shows that Clades A–D are conserved in number across pepper, tomato, and *Arabidopsis*, indicating these subfamilies were present before the *Solanaceae*–*Brassicaceae* split and retained by purifying selection. In contrast, Clades E and F are greatly expanded in pepper (6 and 13 genes) relative to *Arabidopsis* (1–2 genes), implying pepper/tomato-specific duplications. Notably, genes in clusters 2 and 3 (*CaAHL11*–*18* and *CaAHL27*–*29*) and cluster 4 (*CaAHL39*–*42*) all belong to Clades E/F, reinforcing that recent tandem duplication in pepper generated these extra copies. Tandem and proximal duplications are known to provide raw genetic material for the evolution of novel gene functions in plants [26,27], and *CaAHL* genes are no exception. Overall, the *CaAHL* family reflects a blend of ancient conserved regulators and newer, potentially neo-functionalized genes resulting from pepper-specific duplications.

Despite their diversification, CaAHL proteins maintain highly conserved structural features. Almost all members retain the canonical AT-hook and PPC domains (Motifs 1 and 3), underscoring their shared capacity for DNA binding and chromatin interaction [17,26]. The consistent motif architectures and intron–exon patterns within subgroups indicate that duplicated *CaAHL* genes often conserve core functions. At the same time, variable motif insertions or losses in some paralogs (e.g., CaAHL41 lacking Motif 1) hint at functional divergence. The promoters of *CaAHL* genes are rich in hormone- and stress-responsive elements, indicating tight regulation by environmental and developmental signals. For instance, multiple *CaAHL* promoters contain ABA, GA, JA, SA, and anaerobic response elements (Figure 4 and Figure 5). This cis-element diversity suggests that *CaAHL* transcription is modulated by hormonal cues. Consistent with this, related studies have shown *AHL* genes to be hormone-regulated: for example, *Arabidopsis AHLs* influence auxin and gibberellin signaling [11], and rice *AHLs* coregulate flowering and stress pathways [26]. In rice, *OsAHL* genes are markedly upregulated by drought and salinity and form co-expression networks with reproductive genes [26]. Similarly, pepper *CaAHL* promoters harbor stress-related elements, and our expression data show that many *CaAHLs* respond to drought, cold, salt, and oxidative stress. Thus, the conserved domains of *AHL* proteins are embedded in regulatory contexts that link development to environmental adaptation.

The diverse expression profiles of *CaAHL* genes imply extensive sub-functionalization. Many *CaAHLs* are expressed in vegetative tissues: for example, *CaAHL1*, *2*, *5*, *8*, *10*, *20*, *26*, *44* are root/stem-preferential (Figure 6), suggesting roles in root or stem development. Others are fruit/seed-enriched: notably, *CaAHL9*, *27*, *28*, *11*–*18* peak in seeds at 20–25 DAF, and *CaAHL29* spikes only at 60 DAF (Figure 6), indicating possible roles in seed maturation. *CaAHL21* is placenta-enriched (Figure 6), implying involvement in fruit tissue differentiation. In contrast, eight *CaAHL* genes (*12*, *13*, *14*, *16*, *17*, *18*, *27*, *28*) were not expressed in roots even after stress/hormone treatments (Figure 7), aligning with their strictly reproductive expression. Such specialization parallels reported in other species: in rice, nearly all *OsAHLs* were expressed in at least some vegetative or reproductive tissue but with stage-specific peaks [26], while in *Arabidopsis*, different *AHLs* are known to govern distinct developmental processes [5,22]. These patterns suggest that, following duplication, pepper *AHLs* have partitioned developmental roles. Some paralogs, like *CaAHL1* and *CaAHL33*, act broadly in organ development, while others (e.g., *CaAHL29* or *CaAHL36*) are dedicated to specific stages of seed or flower formation.

To further investigate floral development-related candidates, we selected eight *CaAHL* genes that exhibited relatively high transcript levels during flower bud development and validated their expression patterns using qRT-PCR (Figure 8). In this analysis, *CaAHL5* was used as the calibrator gene, and all expression levels of the other *CaAHL* genes were calculated relative to it at each developmental stage. On this basis, *CaAHL4*, *CaAHL35*, *CaAHL33*, *CaAHL23*, and *CaAHL1* consistently exhibited higher expression levels than *CaAHL5* across all stages. *CaAHL26* also showed relatively high expression at most stages, except for F2 and F3. Notably, *CaAHL36* displayed relatively strong expression at early stages (F1 and F2). These expression profiles suggest that certain *CaAHLs* are dynamically regulated during flower bud development and may play diverse roles in this process [8,22]. We also examined the subcellular localization of CaAHL23 and CaAHL36 to gain insight into their possible molecular functions. Both proteins were transiently expressed in *Nicotiana benthamiana* epidermal cells and fused with GFP for visualization. Strong fluorescence signals were observed in the nucleus, with weaker signals in the cytoplasm (Figure 9), indicating that these proteins are primarily nuclear-localized. This localization is consistent with their predicted roles as DNA-binding transcriptional regulators.

The identification of specific *AHLs* associated with flower and anther development provides candidate genes for hybrid breeding. For example, *CaAHL36* could be targeted to create genic male-sterile or -fertile lines, facilitating hybrid seed production. Furthermore, the stress-responsive expression of many *CaAHLs* hints that they may affect stress resilience; breeding or engineering efforts might exploit *AHL* promoters or coding variants to improve tolerance. Future work should test these hypotheses by functional studies, such as CRISPR/Cas9 knockout or overexpression of selected *CaAHLs* to observe effects on fertility and stress tolerance. In conclusion, our genome-wide survey and expression analyses lay a foundation for understanding the roles of *AHL* proteins in pepper biology. The duplication-driven expansion of the *CaAHL* family has allowed pepper to evolve both conserved developmental regulators and novel genes adapted to its specific physiology and environment. This comprehensive resource will enable deeper investigations into how *AHL* transcription factors coordinate plant development and stress responses in *Capsicum*.

## 4. Materials and Methods

### 4.1. Plant Material and Growth Conditions

A highly elite breeding line of pepper (*Capsicum annuum* L.), designated as 6421, was employed in this study. Seeds of 6421 were surface-sterilized with 5% sodium hypochlorite for 15 min, rinsed thoroughly with sterile distilled water, and sown in 200-well seedling trays containing vermiculite. The trays were positioned atop a 30 L lightproof plastic container filled with Japanese garden test nutrient solution (pH 6.0). Seedlings were grown under controlled conditions: a day/night temperature of 25/18 °C, a 16/8 h light/dark cycle, 60–70% relative humidity, and a light intensity of 6000 Lux. Hormone and stress treatments were carried out as previously described [25].

### 4.2. Retrieval and Identification of AHL Genes in Pepper

In this study, the candidate *AHL* proteins were retrieved as follows: first, the protein, nucleotide, and genome sequences of Zhangshugang genomes were downloaded from the Pepper Genomics Database (http://ted.bti.cornell.edu/cgi-bin/pepper/search, accessed on 6 January 2025) [24]. Second, the HMM of *AHL* protein (PF03479) was downloaded from the Pfam database (http://pfam-legacy.xfam.org, accessed on 6 January 2025) [28]. Finally, the TBtools-II software (v2.149) was employed to identify putative *AHL* genes in the Zhangshugang genomes using the “Simple HMM Search” function, with the default cut-off parameters [29]. Specifically, the HMM profile of the *AHL* domain (PF03479) was used as a query, and candidate genes were selected based on the overlap of results in both the “Sequence scores” and “Domain scores” outputs.

### 4.3. Sequence Analysis and Structural Characteristics

The protein lengths, molecular weights, theoretical isoelectric points (pIs), instability indices, and grand average of hydropathicity of CaAHL proteins were analyzed using the TBtools-II (v2.149) software [29]. The supposed subcellular localizations of CaAHL proteins were predicted using the online tool WoLF PSORT (https://wolfpsort.hgc.jp, accessed on 7 January 2025) [30]. The protein sequences were submitted to the MEME program (version 5.5.7, https://meme-suite.org/meme/tools/meme, accessed on 7 January 2025) [31] to assess conserved motifs. The obtained conserved motifs were subjected to functional prediction using the Motif Comparison Tool Tomtom (version 5.5.7, https://meme-suite.org/meme/tools/tomtom, accessed on 8 January 2025) and predicted the potential functions of these motifs.

### 4.4. Chromosome Localization, Tandem Duplication, and Synteny Analysis

Chromosome locations and gene positions in pepper were obtained by searching the Sol Genomics Network. Chromosome mapping of the *CaAHL* gene family was visualized using MG2C (version 2.1, http://mg2c.iask.in/mg2c_v2.1, accessed on 11 January 2025) [32]. Tandem duplication events were further confirmed using the following criteria: (1) the alignment length had a coverage rate of more than 70% of the full length of the *CaAHL* genes; (2) the identity of the aligned region was over 70%; (3) an array of two or more genes was at less than 100 kb distance. TBtools-II (v2.149) software [29] was used to analyze the synteny of *CaAHL* genes across the three pepper genomes.

### 4.5. Phylogenetic Analysis

The phylogenetic tree was generated in the following three steps: first, the CaAHL protein sequences were imported into Clustal X to produce a multiple sequence alignment file. Second, the alignment result was used to build an unrooted tree using MEGA11 with a bootstrap of 1000 replicates and neighbor-joining (NJ) methods [33]. Third, the newly produced phylogenetic tree was visualized using the Interactive Tree of Life online website (version 7.2, https://itol.embl.de, accessed on 10 January 2025) [34].

### 4.6. RNA-Seq Analysis of CaAHL Genes

Transcriptome sequencing (RNA-seq) data for Capsicum line 6421 [25] (http://lifenglab.hzau.edu.cn/PepperHub/index.php, accessed on 5 January 2025) were utilized to investigate the expression profiles of the *CaAHL* gene family across various tissues and developmental stages. The treatment methods for all samples were based on those published by Liu et al. [25]. All data for the *AHL* genes were normalized (log_2_(FPKM+1)), and a heatmap was drawn using TBtools-II (version 2.149) software [29].

### 4.7. RNA Extraction and RT-qPCR Analysis

Total RNA was isolated from floral buds at various developmental stages (F1–F9) using the RNAprep Pure Plant Kit (DP421, TIANGEN, Beijing, China) according to the manufacturer’s instructions. The extracted RNA was then reverse-transcribed into cDNA using the RevertAid First Strand cDNA Synthesis Kit (Thermo Scientific, Waltham, MA, USA). Quantitative real-time PCR (RT-qPCR) was performed on a QuantStudio 3 Real-Time PCR System (Applied Biosystems/Thermo Fisher Scientific, Foster City, CA, USA) using 2×ChamQ Universal SYBR qPCR Master Mix (TransGen, Beijing, China) in accordance with the provided instructions. The primers were designed using Primer-BLAST, a tool available in NCBI (National Center for Biotechnology Information) for finding specific primers (https://www.ncbi.nlm.nih.gov/tools/primer-blast, accessed on 22 January 2025). The *CaActin7* gene was used as the reference gene, with the forward primer sequence 5′-CTCGAGCAGTGTTTCCCAGT-3′ and the reverse primer sequence 5′-AGCTTCATCACCCACATAGGC-3′. Gene-specific primers were designed for *CaAHLs* as follows: *CaAHL36* forward primer, 5′-CAACGTCATCAGGTCGATGT-3′, and reverse primer, 5′-CCAGGTGCCCATGAATAGAC-3′; *CaAHL33* forward primer, 5′-GGAGTTGGCTTTACACCACA-3′, and reverse primer, 5′-TCCATGTGCAGAGAGAATGC-3′; *CaAHL4* forward primer, 5′-TTTTCTTCACGTTGCCTTGTC-3′, and reverse primer, 5′- ATTGGCAGGCATGTTCGATT-3′; *CaAHL23* forward primer, 5′-GAGTCTAGCGGTGGACCTAT-3′, and reverse primer, 5′-AGGTTCTCTCATCTCCAGGG-3′; *CaAHL1* forward primer, 5′-GTCCTACTTCAGGGTCTGGT-3′, and reverse primer, 5′-CCAAATTGTGTCATCCACGC-3′; *CaAHL26* forward primer, 5′-AGGGCTCATTCTAATTGGCG-3′, and reverse primer, 5′-GTGGGACTACTCCACCCTTA-3′; *CaAHL5* forward primer, 5′-CTTCTGGGCTTCCGTTCTTT-3′, and reverse primer, 5′-AAAAGGTGGACGAAGAGCTG-3′; *CaAHL35* forward primer, 5′-CTTGCTGTTTAAAGTTTTCAGTTCT-3′, and reverse primer, 5′-CCTAGAAAAGCCAAAACCCCTA-3′. qRT-PCR thermal cycling conditions were as follows: initial denaturation at 94 °C for 30 s, followed by 43 cycles of denaturation at 94 °C for 5 s and annealing/extension at 60 °C for 30 s. After amplification, a melt curve analysis was performed by heating to 95 °C, then cooling to 60 °C to verify the specificity of each amplicon. RT-qPCR experiments were conducted with three biological replicates and four technical replicates per sample. Relative gene expression levels were determined using the 2^−ΔΔCt^ method.

### 4.8. Subcellular Localization

The full-length ORF sequences of *CaAHL23* or *CaAHL36* without the termination codon were cloned into the pCAMBIA1300-GFP vector and transformed into *Agrobacterium tumefaciens* GV3101. *CaAHL23* or *CaAHL36* fusion constructs were transformed into tobacco (*Nicotiana benthamiana*) leaves. After three days, fluorescence signals were observed and captured using a confocal laser scanning microscope (LSM 510 META, Carl Zeiss, Oberkochen, Germany).

## Figures and Tables

**Table 1:** Basic information on *AHL* genes identified in pepper.

Name	Gene ID	Chromosome Location	Protein Length	Molecular Weight (kDa)	Theoretical pI	Instability Index	Grand Average of Hydropathicity	Subcellular Localization ^1^
CaAHL1	Caz01g03310.1	Chr01:5691094–5697294	347	35.27	9.62	38.59	−0.33	nucl
CaAHL2	Caz01g04670.1	Chr01:9114966–9116603	295	31.17	5.79	52.67	−0.54	nucl
CaAHL3	Caz01g04810.1	Chr01:9383031–9386809	322	33.18	8.74	50.79	−0.17	chlo
CaAHL4	Caz01g08370.1	Chr01:20063956–20073612	418	43.66	8.82	60.17	−0.76	nucl
CaAHL5	Caz01g08400.1	Chr01:20111560–20112474	304	31.69	5.75	51.83	−0.27	nucl
CaAHL6	Caz01g13980.1	Chr01:55893363–55894391	314	33.10	6.83	53.54	−0.60	nucl
CaAHL7	Caz01g14080.1	Chr01:56075436–56079692	324	33.72	9.57	47.83	−0.25	chlo
CaAHL8	Caz01g31040.1	Chr01:251924071–251924907	278	29.43	6.54	50.25	−0.47	chlo
CaAHL9	Caz01g35190.1	Chr01:276409484–276418256	123	13.45	5.87	47.09	−0.20	cyto
CaAHL10	Caz01g40510.1	Chr01:326409380–326414186	265	28.25	5.96	44.58	−0.44	nucl
CaAHL11	Caz01g40520.1	Chr01:326434617–326442536	331	35.20	9.51	49.49	−0.46	chlo
CaAHL12	Caz01g40530.1	Chr01:326492721–326618469	316	33.80	10.04	51.86	−0.37	nucl
CaAHL13	Caz01g40540.1	Chr01:326621531–326626277	207	22.30	6.96	44.75	−0.13	nucl
CaAHL14	Caz01g40550.1	Chr01:326687022–326691770	207	22.34	7.84	44.38	−0.14	chlo
CaAHL15	Caz01g40640.1	Chr01:327024609–327031508	329	34.72	9.65	48.09	−0.47	chlo
CaAHL16	Caz01g40680.1	Chr01:327253871–327258557	288	30.70	8.76	47.03	−0.45	chlo
CaAHL17	Caz01g40710.1	Chr01:327378313–327383011	273	29.34	7.79	47.92	−0.53	chlo
CaAHL18	Caz01g40720.1	Chr01:327501110–327505809	273	29.30	7.79	51.24	−0.53	chlo
CaAHL19	Caz01g41290.1	Chr01:329720440–329727085	332	34.13	9.54	44.92	−0.10	chlo
CaAHL20	Caz01g41410.1	Chr01:329900501–329901727	316	33.79	6.05	59.8	−0.63	chlo
CaAHL21	Caz01g41740.1	Chr01:331181444–331186914	352	35.75	9.44	49.49	−0.27	nucl
CaAHL22	Caz02g02600.1	Chr02:44359225–44368231	345	34.69	8.97	55.44	−0.11	E.R.
CaAHL23	Caz02g20690.1	Chr02:156842236–156848762	331	33.33	9.99	50.43	−0.23	nucl
CaAHL24	Caz03g00210.1	Chr03:551605–558146	111	12.02	6.39	30.43	0.01	chlo
CaAHL25	Caz03g21400.1	Chr03:72579177–72587401	346	36.17	6.33	45.59	−0.38	nucl
CaAHL26	Caz03g34730.1	Chr03:267518820–267519650	276	28.85	5.45	57.84	−0.47	cyto
CaAHL27	Caz03g36660.1	Chr03:273808903–273810928	122	12.87	5.19	34.02	0.18	cyto
CaAHL28	Caz03g36670.1	Chr03:273824211–273825851	113	12.01	6.01	47.05	−0.25	nucl
CaAHL29	Caz03g36680.1	Chr03:273829352–273850851	273	28.76	9.64	49.08	−0.30	vacu
CaAHL30	Caz04g00390.1	Chr04:713251–718475	267	26.36	6.42	48.45	−0.13	nucl
CaAHL31	Caz04g08200.1	Chr04:20375417–20376564	352	37.79	7.05	63.62	−0.66	nucl
CaAHL32	Caz05g17580.1	Chr05:235719249–235723745	294	31.56	5.35	52.68	−0.61	nucl
CaAHL33	Caz06g17080.1	Chr06:50064634–50076684	349	36.50	9.34	53.47	−0.51	nucl
CaAHL34	Caz06g17990.1	Chr06:57415662–57416120	152	15.59	4.44	51.44	−0.03	nucl
CaAHL35	Caz06g24080.1	Chr06:182665460–182672828	341	35.70	7.02	40.07	−0.30	plas
CaAHL36	Caz07g19270.1	Chr07:251735483–251736292	269	28.24	9.24	33.67	−0.13	cyto
CaAHL37	Caz08g07460.1	Chr08:137932240–137938844	341	34.99	10.26	58.12	−0.22	nucl
CaAHL38	Caz09g21720.1	Chr09:274809109–274813725	438	45.01	9.38	50.94	−0.34	nucl
CaAHL39	Caz12g05920.1	Chr12:13682382–13697371	578	60.51	7.72	52.36	−0.30	chlo
CaAHL40	Caz12g05950.1	Chr12:13811174–13813071	115	11.71	11.25	59.27	−0.01	chlo
CaAHL41	Caz12g06070.1	Chr12:14037114–14041618	177	18.10	4.89	59.53	−0.20	chlo
CaAHL42	Caz12g06080.1	Chr12:14056732–14062760	438	47.47	9.27	50.27	−0.21	chlo
CaAHL43	Caz12g08880.1	Chr12:32039622–32040395	257	28.05	7.83	49.44	−0.42	nucl
CaAHL44	Caz12g18220.1	Chr12:219722263–219725994	293	29.60	6.16	46.17	−0.33	nucl
CaAHL45	Caz12g18510.1	Chr12:221530027–221540499	358	37.13	9.57	50.03	−0.37	cyto

^1^ Note: nucl, nucleus; chlo, chloroplast; cyto, cytoplasm; plas, plasma membrane; vacu, vacuole membrane; E.R., endoplasmic reticulum.

## Data Availability

Data are contained within the article and Supplementary Materials.

## Supplementary Materials

The following supporting information can be downloaded at https://www.mdpi.com/article/10.3390/ijms26136527/s1.
